# STRATEGIES FOR OLDER ADULT AND INFORMAL CAREGIVER INVOLVEMENT IN HEALTH POLICY DEVELOPMENT: STAKEHOLDERS’ VIEWS

**DOI:** 10.1093/geroni/igad104.1609

**Published:** 2023-12-21

**Authors:** Opeyemi Kolade, Joshua Porat-Dahlerbruch, Saritte Perlman, Theo van Achterberg, Moriah Ellen, Opeyemi Kolade, Joshua Porat-Dahlerbruch, Saritte Perlman, Theo van Achterberg, Moriah Ellen

**Affiliations:** Ben-Gurion University of the Negev, Midreshet, HaDarom, Israel; University of Pittsburgh, Pittsburgh, Pennsylvania, United States; Ben Gurion University of the Negev, Beersheba, Israel; KU Leuven, Leuven, Vlaams-Brabant, Belgium; Ben Gurion University of the Negev, Beer Sheva, HaDarom, Israel; Ben-Gurion University of the Negev, Midreshet, HaDarom, Israel; University of Pittsburgh, Pittsburgh, Pennsylvania, United States; Ben Gurion University of the Negev, Beersheba, Israel; KU Leuven, Leuven, Vlaams-Brabant, Belgium; Ben Gurion University of the Negev, Beer Sheva, HaDarom, Israel

## Abstract

Older adults and informal caregivers’ involvement in policy-making processes can enhance the creation of responsive policies and the provision of better health and well-being services. However, current involvement literature is more focused on involvement in research and healthcare decision-making. This research aims to explore stakeholders’ perspectives on approaches for the involvement of older adults and informal caregivers in health policy development. A qualitative study using semi-structured interviews was conducted within the European TRANS-SENIOR consortium and included policymakers, researchers, and advocacy organizations in nine countries. Participants were included if they had experience with older adult/informal caregiver involvement in health policy development. A total of eighteen interviews were conducted with nine representatives from older adult/informal caregiver advocacy organizations, five policymakers, and four researchers. Identified themes were: 1) diversity of involvement approaches, 2) impact of involving older adults and informal caregivers in health policy development, and 3) facilitators and barriers of involvement. Findings indicated that older adult and informal caregiver involvement vary in nature and intensity and that the actual effect of older adult and informal caregiver involvement in health policy development is difficult to assess. There is no one-size-fits-all approach for involving older adults and informal caregivers in health policy development; stakeholders can focus on exploring and implementing context-specific tailored strategies. Our study findings provide evidence that can be useful to decision-makers to involve older adults and informal caregivers in health policy development.

